# Efficacy and safety of thread embedding acupuncture combined with conventional acupuncture for chronic low back pain: A study protocol for a randomized, controlled, assessor-blinded, multicenter clinical trial

**DOI:** 10.1097/MD.0000000000010790

**Published:** 2018-05-25

**Authors:** Jungtae Leem, Hyunho Kim, Hee-Geun Jo, Sae-rom Jeon, Yejin Hong, Yeoncheol Park, Byungkwan Seo, Yeeun Cho, Jung Won Kang, Eun-Jung Kim, Ga Young Han, Jae Soo Kim, Hyun-Jong Lee, Tae-Hun Kim, Dongwoo Nam

**Affiliations:** a Dongshin Korean Medicine Hospital, Seoul; b Chung-Yeon Medical Institute; c Chung-Yeon Korean Medicine Hospital, Gwangju; d Department of Clinical Korean Medicine; e Department of Acupuncture & Moxibustion, Graduate School; f Department of Acupuncture & Moxibustion, College of Korean Medicine, Kyung Hee University, Seoul; g Department of Acupuncture & Moxibustion, Dongguk University Bundang Oriental Hospital, Gyeonggi-do; h Department of Acupuncture & Moxibustion, College of Korean Medicine, Daegu Haany University, Gyeongsangbuk-do; i Department of Clinical Research of Korean Medicine, Kyung Hee University, Seoul, South Korea.

**Keywords:** acupuncture, catgut, low back pain, randomized controlled trial, study protocol, thread embedding acupuncture

## Abstract

**Background:**

Back pain is one of the most common diseases, and many patients with recurrent pain seek alternative treatment strategies. Thread embedding acupuncture involves thread insertion at the acupuncture point for continuous physical and chemical stimulation. Although thread embedding is widely used in clinical practice, there is no sound evidence of its efficacy for chronic back pain. We describe the protocol for a randomized controlled trial for investigation of the efficacy and safety of thread embedding acupuncture combined with conventional acupuncture for chronic low back pain.

**Methods:**

This randomized, controlled, assessor-blinded, 2-armed, parallel, multicenter clinical trial will include 38 outpatients with chronic low back pain recruited from 4 traditional Korean Medicine hospitals. The patients will be randomly allocated to a treatment group (conventional acupuncture + thread embedding acupuncture) and a control group (only conventional acupuncture) in a 1:1 ratio. The treatment group patients will receive thread embedding acupuncture treatment at 10 acupuncture points (multifidus muscle, 4 points; spinal erector muscles, four points; lumbar quadrate muscle, 2 points) once a week for 8 weeks (8 sessions). In addition, all patients will receive conventional acupuncture treatment at 14 acupuncture points (GV3, EX-B5, and bilateral BL23, BL24, BL25, BL26, BL40, and BL60) twice a week for 8 weeks (16 sessions). The primary outcome will be the change in the visual analog scale score from visit 1 to visit 16, analyzed by independent *t* tests, in both groups. The groups will also be compared with regard to the clinical relevance (minimal clinically important difference), quality of life (3-level version of Euroqol-5D), disability level (Roland and Morris Disability Questionnaire), global assessment (patient global impression of change), and safety. Cost data for cost–benefit and cost-effectiveness analyses will be collected.

**Discussion:**

Our study results will provide evidence of the efficacy and safety of thread embedding acupuncture combined with conventional acupuncture for the management of chronic low back pain. Even though the assessors will be blinded, the patients will not be blinded to treatment because of the lack of a sham embedding acupuncture group; this is a limitation of our study.

**Trial registration:**

Clinical Research Information Service: KCT0002666

## 1 Background

In developed countries, more than 70% individuals experience low back pain. Approximately 90% cases of back pain are nonspecific with an unclear pathophysiological origin.^[1,2]^ Back pain is classified as acute, subacute, and chronic according to the pain duration. Conventionally, pain for <6 weeks, 6 to 12 weeks, and >12 weeks is categorized as acute pain, subacute pain, and chronic pain, respectively.^[3]^ Back pain easily recurs without proper treatment and rehabilitation. Psychological factors also prolong back pain.^[4]^ In a cohort of patients with chronic low back pain refractory to general conservative therapy, pain was associated with the facet joint of the spine in 31% cases, sacroiliac joint in 18% cases, and spinal discs in 42% cases.^[5]^ Although conventional western medicine treatment has shown efficacy for the treatment of back pain,^[6]^ several patients still experience pain and require alternative treatment strategies.^[7]^ In fact, the number of patients opting for complementary and alternative medicine (CAM) is gradually increasing.^[8]^ Acupuncture, moxibustion, chiropractic manipulation, massage, yoga, Tai chi, and Qigong are frequently used CAM therapies.^[7,8]^

Thread embedding acupuncture (TEA), a type of dermal needle insertion therapy, involves the insertion of thread at specific points for the treatment of various disorders.^[9]^ Because of sustained stimulation, TEA has been used to treat chronic diseases.^[10]^ TEA provides both physical and chemical stimulation. The former refers to continuous acupuncture point stimulation after thread insertion, while the latter refers to tissue damage caused by thread insertion, which results in an aseptic inflammatory reaction and ultimately promotes tissue regeneration.^[11]^ In the early days, TEA was used to treat obesity for cosmetic purposes. However, it is currently used to treat several musculoskeletal conditions such as ankle sprain,^[12]^ shoulder pain,^[13]^ lumbar intervertebral disc herniation,^[14]^ and plantar fasciitis.^[15]^

In cases of chronic back pain, if the treatment frequency is not maintained for a certain regular period, the success rate decreases and the risk of recurrence increases. TEA stimulates the surrounding spinal muscles and ligaments, while the thread dissolves and promotes the recovery and strengthening of the ligaments and muscles around the waist. Sustained effects of infiltration and the promotion of tissue regeneration enhance the pain-relieving effects. However, most published studies have been case reports, and well-designed randomized controlled clinical trials are necessary to confirm the efficacy and safety of TEA for chronic low back pain. Here, we describe the protocol for a randomized controlled clinical trial for investigation of the efficacy and safety of TEA combined with conventional acupuncture for chronic low back pain via assessments of the pain intensity, disability level, clinical relevance, quality of life, global impression change, and safety outcomes.

## 2 Methods/design

This study protocol followed the Standard Protocol Items: Recommendations for Interventional Trials (SPIRIT) checklist, which enhanced the quality of the clinical trial.^[16]^

### 2.1 Objectives

The aim of this study was to examine the efficacy and safety of TEA combined with conventional acupuncture for chronic low back pain through comparisons with the effects of conventional acupuncture alone

### 2.2 Trial design and study setting

**Figure 1:**
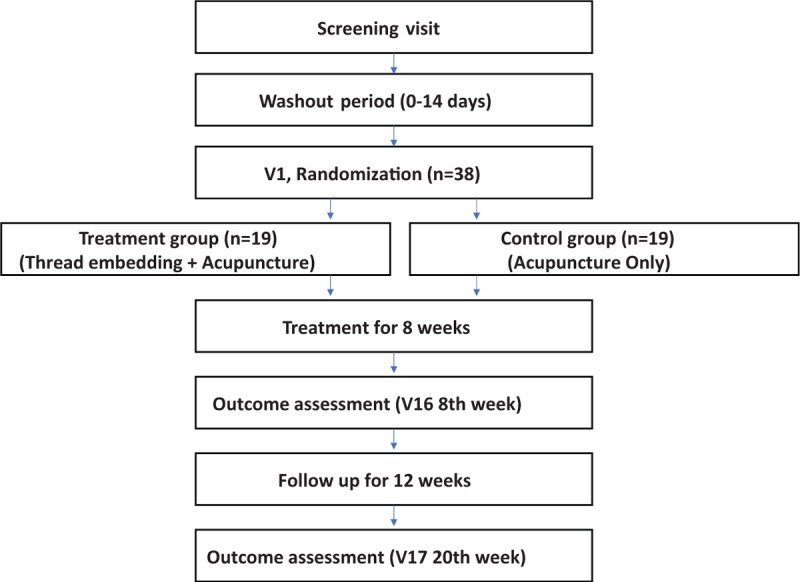
Study flowchart.

The study is a randomized, sham-controlled, parallel group, assessor-blinded, multicenter, superiority clinical trial. Thirty-eight participants who meet the eligibility criteria will be randomly assigned to a treatment group (TEA + conventional acupuncture) or a control group (only conventional acupuncture) in a 1:1 ratio. The trial design and study flowchart are shown in Fig. 1.

### 2.3 Recruitment

Four Korean Medicine hospitals located in the Republic of Korea, namely Kyung Hee University Korean Medicine Hospital, Kyung Hee University Korean Medicine Hospital at Gangdong, Oriental Medicine Hospital of Daegu Haany University, and Dongguk University Bundang Oriental Hospital, will recruit outpatients in the clinical practice setting. Clinical trial information will be posted on the bulletin board of each hospital and university, in local newspapers, in online advertisements, and on advertisement boards in public spaces.

### 2.4 Eligibility criteria: inclusion criteria

1. Male and female subjects aged 19 to 65 years;
2. Chief complaint of low back pain for >3 months;
3. Pain and discomfort rated beyond 40 mm on a 100-mm visual analog scale (VAS) for 1 week before enrollment;
4. Voluntary provision of written informed consent and willingness to follow trial-related instructions.

### 2.5 Eligibility criteria: exclusion criteria

1. History of hypersensitive reactions to TEA or conventional acupuncture;
2. Abnormal signs in neurological examinations of the lower extremity;
3. Previous surgical treatment for severe neurological deficits (motor or sensory) or cauda equine syndrome;
4. History of spinal surgery or scheduled spinal surgery during the clinical trial period
5. Neuromuscular scoliosis or neurodegenerative disease;
6. Diseases that can cause secondary back pain, such as vertebral fracture, inflammatory spondylitis, spinal infection, and malignant tumor;
7. Pregnancy, lactation, plans to conceive, or refusal to use appropriate contraception methods during the trial period;
8. History of acupuncture, medication, physical therapy, or manipulation therapy for back pain within the last 2 weeks;
9. Use of anticoagulants;
10. More severe pain in areas other than the lumbar region;
11. Ineligibility for the trial judged by the clinical trial investigator.

### 2.6 Subject withdrawal criteria

1. Violation of the inclusion criteria or fulfillment of the exclusion criteria;
2. Serious adverse events that impede trial continuation;
3. Withdrawal of consent by the subject or a legal representative;
4. Do not receive 3 consecutive treatment sessions or receipt of <6 TEA sessions;
5. Violation of the clinical trial protocol by the investigator or the subject;
6. Loss to follow-up;
7. Use of medications or treatments that can affect the results of the clinical trial without permission from the investigator;
8. Inappropriate progress as judged by the investigator.

### 2.7 Randomization and allocation concealment

A randomization table for each hospital will be created by an independent statistician using the block randomization method with SAS Version 9.4 (SAS institute. Inc., Cary, NC). The randomization number will be sealed in a sequentially numbered opaque envelope and sent to each hospital. The random number envelope will be kept in a double locked cabinet. Consent will be obtained by a Korean Medicine doctor. If the participant voluntarily signs informed consent and meets the inclusion criteria, the clinical research coordinator (CRC) will sequentially give the sealed random number envelope to the physician, who will open the envelope and allocate the participant to the treatment or control group according to the random number.

### 2.8 Blinding

Because of the add-on study design, the participants and practitioners cannot be blinded. The assessor will be blinded. The treating physician will not assess the outcome measures. The assessor will be instructed to refrain from talking about the treatment in order to maintain blinding. The statistician and data collector will also be blinded.

### 2.9 Intervention

#### 2.9.1 Study schedule

The study schedule is presented in Table 1. The trial will include a screening phase, treatment phase, and follow-up phase. At the screening visit, participants will sign the informed consent form. The investigator will perform a physical examination, record the medical history, obtain an L-spine X-ray, collect blood samples, record any concomitant treatment history, and use VAS to determine whether the participant is eligible for the clinical trial. If the participant is taking anti-inflammatory drugs or pain killers, we will implement a 2-week washout period. During the 8 weeks of treatment, the patient will visit the facility twice a week (16 visits). Conventional acupuncture treatment will be administered at every visit in both the treatment group and the control group. Eight sessions of TEA will be conducted once a week only in the treatment group (visits 1, 3, 5, 7, 9, 11, 13, 15). On visits 17, the participants will visit the facility 12 weeks after visit 16. VAS scores for pain will be determined on visits 1, 4 (week 2), 8 (week 4), 12 (week 6), 16 (week 8), and 17 (week 20).

**Table 1:**
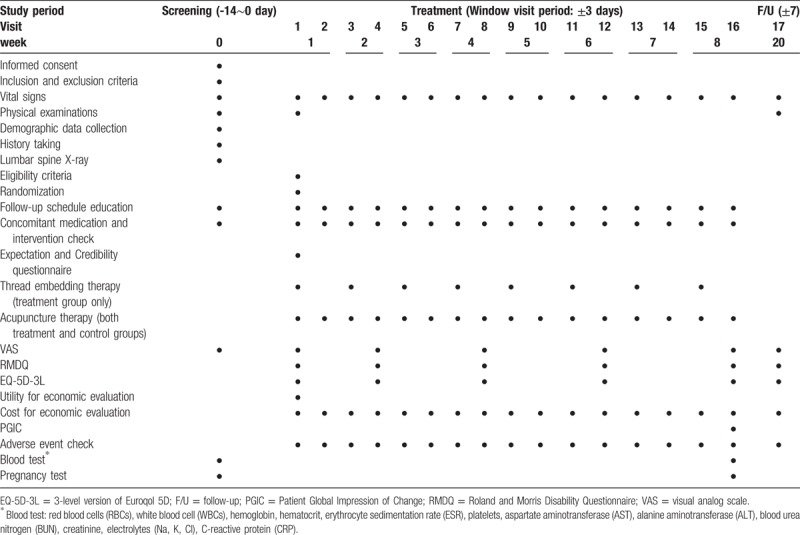
Study schedule.

#### 2.9.2 Treatment group intervention

**Figure 2:**
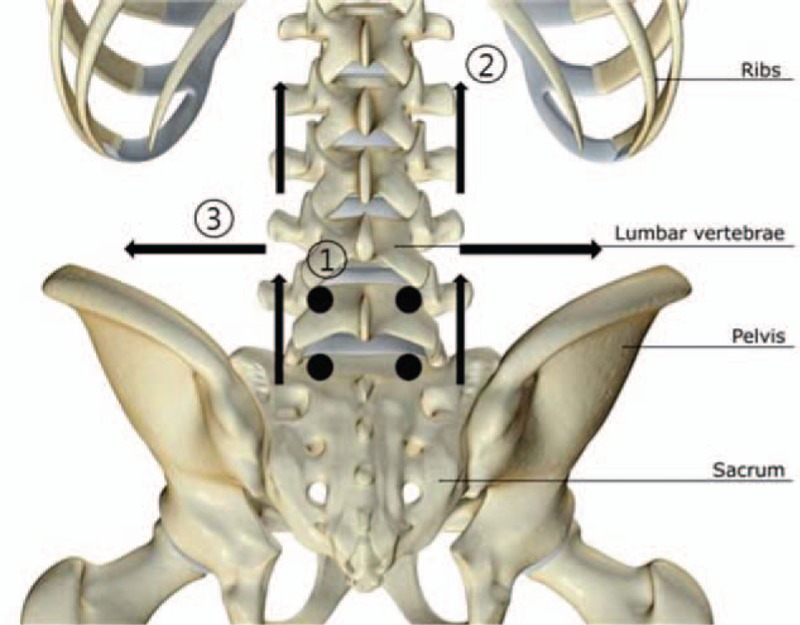
Point location and direction of thread embedding acupuncture for patients with chronic low back pain. (1) Multifidus muscle: Perpendicular insertion at EX-B2 points positioned on either side of L4–5 (n = 2) and L5-S1 (n = 2). (2) Spinal erector muscles: Transverse insertion at points located 3–4 cm from the L3 (one on each side, n = 2) and S1 spinous processes (one on each side, n = 2), toward L1. The thread will be located in the shallow muscle layers. (3) Lumbar quadrate muscle: Transverse insertion at 2 points, from the L4 spinous process toward the bilateral iliac crest. The thread will be located in the shallow muscle layers.

TEA will be applied to the muscles in the low back area using a 29-gauge, 40-mm polydioxanone suture (Hyundae Meditech Co., Weonju, South Korea). A total of 10 points will be assigned to each subject. For the multifidus muscle, the thread will be inserted in a perpendicular direction at EX-B2 points positioned on either side of L4–5 (n = 2) and L5-S1 (n = 2). For the spinal erector muscles, the thread will be inserted in a transverse direction at points located 3 to 4 cm away from the L3 (one on each side, n = 2) and S1 (one on each side, n = 2) spinal processes, toward L1. The thread will be located in the shallow muscle layers. For the lumbar quadrate muscle, the thread will be inserted in a transverse direction at 2 points, from the L4 spinous process toward the bilateral iliac crest. The thread will be located in the shallow muscle layers (Fig. 2).

In the treatment group, conventional acupuncture treatment will be administered immediately after TEA. Sterilized stainless steel needles (DB108C; Dongbang Medical Co., Boryung-si, South Korea) measuring 0.25 mm in width and 40 mm in length will be inserted at 10 local and 4 distal acupuncture points. The 10 local acupuncture points are as follows: GV3, EX-B5, and bilateral BL23, BL24, BL25, and BL26. The 4 distal acupuncture points are as follows: bilateral BL40 and BL60. Details about the treatment group intervention are described in the Standards for Reporting Interventions in Clinical Trials of Acupuncture (STRICTA) checklist in Table 2.^[17]^

**Table 2:**
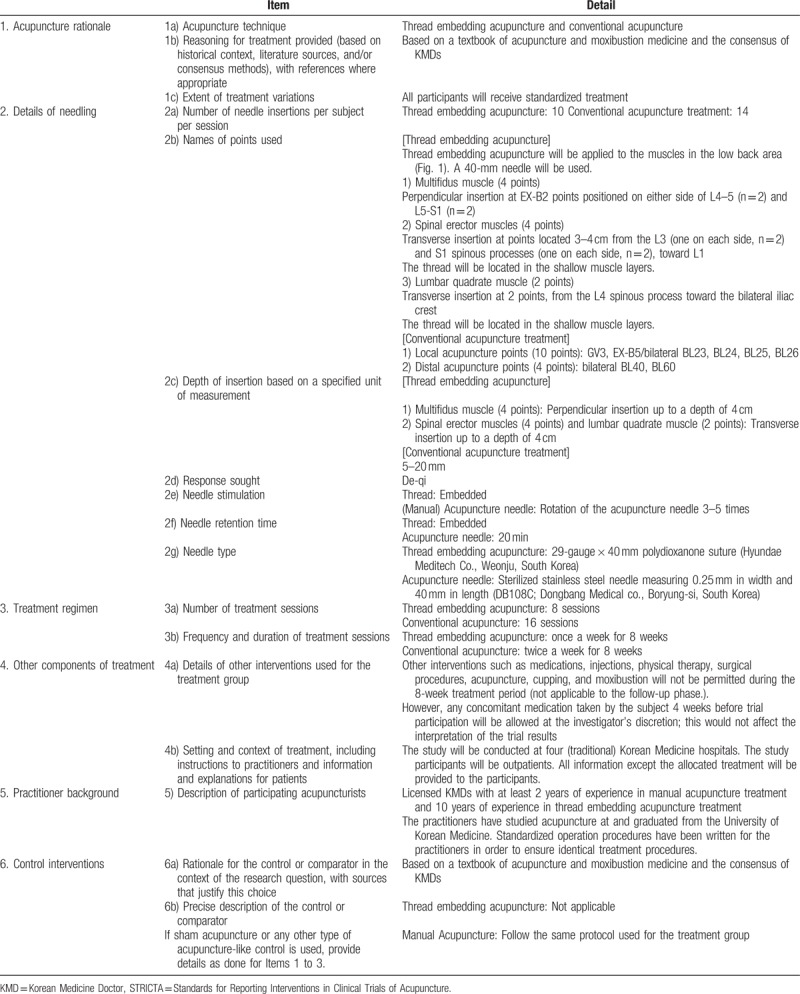
Details of acupuncture treatments for chronic low back pain, based on the STRICTA 2010 checklist.

#### 2.9.3 Control group intervention

The control group patients will receive conventional acupuncture treatment following the same protocol used for the treatment group. TEA will not be administered.

#### 2.9.4 Concomitant treatment

Other interventions such as medications, injections, physical therapy, surgical procedures, acupuncture, cupping, and moxibustion will not be permitted during the 8-week treatment period (not applicable to the follow-up phase). However, any concomitant medications taken by the subject within 4 weeks before trial participation, which will not affect the interpretation of the trial outcomes, will be allowed at the discretion of the investigator.

### 2.10 Outcome measures: primary endpoint

The primary outcome of our study will be the change in the VAS score for pain from baseline (visit 1) to treatment completion (week 8, visit 16).^[18]^ The patient will quantify the pain intensity by marking the most relevant point on a 100-mm horizontal line anchored by 2 descriptors: 100, representing the “worst imaginable pain,” and 0, representing “no pain.”

### 2.11 Outcome measures: secondary endpoints

#### 2.11.1 Pain

Every change in the VAS score between baseline (visit 1) and week 2 (visit 4), week 4 (visit 8), week 6 (visit 12), and 3 months after treatment completion (visit 17).

#### 2.11.2 Clinical relevance

The minimal clinical important difference (MCID) in the VAS score for chronic low back pain was defined as a decrease of 20 mm.^[19]^ The proportion of patients showing a VAS score decrease of >20 mm (MCID) was compared between the treatment and control groups at week 2 (visit 4), week 4 (visit 8), week 6 (visit 12), week 8 (visit 16), and 3 months after treatment completion (visit 17). The proportion of patients with a VAS score decrease of >30% and >50% relative to the baseline score will also be compared.

#### 2.11.3 Disability level

The Korean version of the Roland and Morris Disability Questionnaire (RMDQ) will be used to assess disability due to back pain.^[20]^ The change in the RMDQ score at week 2 (visit 4), week 4 (visit 8), week 6 (visit 12), week 8 (visit 16), and 3 months after treatment completion (visit 17) will be compared between the 2 groups. RMDQ comprises 24 questionnaires, and a higher score indicates greater disability.

#### 2.11.4 Quality of life

The 3-level version of Euroqol-5D (EQ-5D-3L) will be used to assess the quality of life.^[20]^ The change in the EQ-5D-3L score at week 2 (visit 4), week 4 (visit 8), week 6 (visit 12), week 8 (visit 16), and 3 months after treatment completion (visit 17) will be compared between the 2 groups. The patients will rate their quality of life in 5 categories using a 3-point scale.

#### 2.11.5 Global assessment

The Patient Global Impression of Change (PGIC) score will be used for the global assessment of back pain. PGIC assesses the perceived health status of patients on a 7-point Likert scale ranging from 1 (very much worse) to 7 (very much improved). Grade 4 represents no change.^[21]^ The change in the PGIC score at week 2 (visit 4), week 4 (visit 8), week 6 (visit 12), week 8 (visit 16), and 3 months after treatment completion (visit 17) will be compared between groups.

#### 2.11.6 Economic analysis

Cost–benefit and cost-effectiveness studies will be simultaneously conducted. Cost data will be collected using a separate economic analysis case report form (CRF). The medical cost will include the official medical cost (paid by the patient to the hospital) and the unofficial medical cost, which includes the cost of over-the-counter drugs, health foods, medical devices, and other requirements. The nonmedical cost includes the transportation cost, care cost, and time cost. The work productivity lost will also be assessed.

#### 2.11.7 Credibility test

The credibility of TEA will be assessed using the Credibility/Expectancy questionnaire at the screening visit. It assesses the credibility and expectancy of the treatment on a 9-point numeric rating scale, with a higher score indicating higher expectancy and credibility.^[22]^

### 2.12 Safety assessment

Physical examinations will be performed and vital signs and adverse events will be checked at every visit. A pregnancy test will be conducted at the screening visit. To examine the safety of TEA, a blood test will be conducted at visit 1 and visit 16. The red blood cell (RBC) count, white blood cell (WBC) count, hemoglobin level, hematocrit value, erythrocyte sedimentation rate (ESR), platelet count, aspartate aminotransferase (AST) level, alanine aminotransferase (ALT) level, blood urea nitrogen (BUN) level, creatinine level, electrolyte (Na, K, Cl) level, and C-reactive protein (CRP) level will be assessed. Adverse events will be assessed according to the Standardizing Assessment and Reporting of Adverse Effects in Rheumatology Clinical Trials II: The Rheumatology Common Toxicity Criteria v.2.0. (RCTC v.2.0).^[23]^ Any adverse event during the clinical trial will be recorded and assessed in CRF. The adverse event occurrence ratio will be calculated. Adverse events associated with the intervention will be monitored and appropriately handled.

### 2.13 Data management and quality control

CRC will collect data on a paper CRF. Data will be collected according to a preapproved study protocol. To maintain confidentiality, all original source documents, including medical records, questionnaires, consent forms, and other relevant records will be stored at each site in a locked cabinet or a password-protected computer that can be accessed only by an authorized individual. The data will be stored for 3 years after study completion.

Any protocol revision will be handled by Kyung Hee University Korean Medicine Hospital, which will serve as a central coordinating facility. An independent clinical research associate (CRA) will conduct monitoring at each site and ensure that the rights of the subjects are protected, and the clinical trial is conducted in accordance with the research protocol. CRA will monitor the data quality, protocol compliance, informed consent forms, recruitment status, data collection, CRFs, and other relevant documents and processes. Regular audits will be conducted by the Ministry of Food & Drug Safety (MFDS) in Korea. All practitioners and investigators have gone through a good clinical practice training course. To enhance the quality of TEA, it will be performed by a practitioner with >10 years of experience.

### 2.14 Sample size calculation

Sample size calculation was performed to determine the appropriate number of participants. The primary outcome will be the change in the VAS score from visit 1 to visit 16 in the treatment and control groups. To our knowledge, no study has investigated the effects of TEA combined with conventional acupuncture treatment. A previous clinical trial evaluated the effects of TEA and sham TEA administered for 4 weeks.^[24]^ The change in the VAS score after treatment (week 4) was 1.85 ± 0.86 in the treatment group and 0.5 ± 0.80 in the control group. The required number of participants per group was 11, considering a 2-tailed test with a 1:1 allocation ratio, a superiority trial, a test power of 95% (1-β), and a significance level of 5% (α). After considering a 40% dropout rate, we calculated the sample size for our study to be 19 patients per group. Thus, a total of 38 participants will be required.

### 2.15 Statistical analysis

Data will be expressed as means and standard deviations for continuous variables and frequencies and ratios (%) for categorical variables. The patients’ baseline characteristics will be compared using an independent *t* test or the Wilcoxon rank-sum test for continuous variables and a χ^2^ test or Fisher exact test for categorical variables. If there is a significant difference in the baseline characteristics between groups, analysis of covariance will be used for efficacy analysis, which will include both a full analysis set (FAS) and a per-protocol set (PPS). FAS will be used for the main analysis and PPS will be used for subanalysis of sensitivity. FAS will include patients who will receive at least 1 session of treatment and will undergo at least 1 pain assessment using VAS after treatment. FAS will not include participants who do not meet the eligibility criteria, those who do not receive any treatment, or those whose data are not acquired after randomization. PPS will include patients who will receive at least 12 sessions of treatment (75% compliance rate), those who undergo pain assessment using VAS at visit 16, and those who will complete the clinical trial without major protocol violation. In the primary outcome analysis, the change in the VAS score from visit 1 to visit 16 will be compared between the treatment group and the control group using an independent *t* test or the Wilcoxon rank-sum test according to normality. In the secondary outcome analysis of continuous variables, a *t* test or the Wilcoxon rank-sum test will be applied according to normality. In the secondary outcome analysis of categorical variables, a χ^2^ test or Fisher exact test will be applied. In safety analysis, the total adverse event ratio and adverse event ratio associated with TEA will be compared using a χ^2^ test or Fisher exact test. The last observation carried forward method will be used for missing data. The level of significance will be 5% (2-tailed test). No correction method will be applied for multiple comparisons in the secondary outcome analysis.

### 2.16 Ethics approval and registration

This study was approved by the Institutional Review Board of Kyung Hee University Korean Medicine Hospital (KOMCIRB-160919-HR-050-13), Kyung Hee University Korean Medicine Hospital at Gangdong (KHNMC OH 2016-10-012), Oriental Medicine Hospital of Daegu Haany University (DHUMC-D-16013-ANS-02), and Dongguk University Bundang Oriental Hospital (DUBOH-IRB-2016-0013). Written informed consent will be obtained from all participants. All participants will have enough time to decide whether they would like to participate in the study. Their confidentiality will be protected by the study identification number. A subject identification log containing personal information about the patient will be kept in a locked cabinet that only an authorized person can access. This study protocol is registered in Clinical Research Information Service (CRIS; https://cris.nih.go.kr). The clinical trial registration number is KCT0002666. The final protocol version is 2.21 and dated July 26, 2017.

## 3 Discussion

Back pain is the most common musculoskeletal condition experienced during the lifetime of approximately 75% adults.^[25]^ Even though musculoskeletal pain is well controlled by conventional treatments such as nonsteroidal anti-inflammatory drugs, the long-term use of medication is not recommended because of several side effects.^[25]^ Patients with chronic low back pain also exhibit a poorer quality of life than do healthy individuals.^[26]^ Because many patients are not satisfied with conventional medicine treatment,^[7]^ the demand for CAM is only increasing.

TEA involves the insertion of polydioxanone sutures into specific regions such as muscles or subcutaneous tissues located at the acupuncture point.^[10]^ Polydioxanone is a synthetic monofilamentous polymer made of polyester or a polydioxanone polymer.^[27]^ TEA is widely used in Korea, Taiwan, and China.^[26]^ In China, TEA is frequently used in internal medicine departments for the management of conditions such as epigastric pain and obesity. Surgical departments also frequently use TEA for the management of low back pain and leg pain, while dermatology departments frequently use it for the management of conditions such as psoriasis. The success rate for TEA used for surgical and dermatological applications is more than 90% in Chinese studies.^[28]^ In Korea, TEA is also used for facial rejuvenation in cosmetic clinics^[27]^ and for the treatment of back pain. In a retrospective chart review, TEA combined with conventional acupuncture showed a better improvement in Numerical Rating Scale and Oswerty Disability Index scores.^[29]^ The physiological and chemical effects of the embedded thread soften the subcutaneous tissue and muscle, resulting in gradual dissolution of the thread.^[30]^ However, well-designed clinical trials on the safety and efficacy of TEA for chronic low back pain do not exist.^[31]^ Therefore, this trial is expected to provide evidence concerning the usefulness of TEA for this patient population.

This study has several strengths. First, experienced practitioners will provide TEA treatment, thus enhancing the quality of trial data. Second, the multicenter setting will prevent bias. Third, we will evaluate not only the pain intensity but also the clinical relevance, quality of life, disability level, global assessment, and costs. Fourth, we will examine the effects of TEA combined with conventional acupuncture, which reflects an actual clinical setting. However, several limitations also exist. First, we calculated the sample size on the basis of a study with a protocol different from the presently described one; the treatment duration in the previous study was only 4 weeks,^[24]^ whereas that in our study is 8 weeks. Therefore, we could have overestimated the sample size. Second, the previous study^[24]^ compared actual TEA with sham TEA; participants were accordingly blinded to the allocated group. In this study, no TEA will be used in the control group, so the participants and practitioners will not be blinded to the allocated group. This could influence the results of the study. Finally, we have not specified any rescue drugs, which could result in an increased dropout rate due to severe pain. Nevertheless, the study design is ideal to determine the precise therapeutic effects of TEA. Despite the limitations, the findings of this trial are expected to provide evidence about the efficacy and safety of TEA for chronic low back pain, which will be useful for physicians, stakeholders, patients, and researchers.

### 3.1 Trial status

This trial is currently recruiting participants. Recruitment began on September 12, 2017. We expect the recruitment phase to be complete by the end of 2018.

## Author contributions

**Conceptualization:** Eun-Jung Kim, Jae Soo Kim, Hyun-Jong Lee.

**Funding acquisition:** Dongwoo Nam.

**Investigation:** Sae-rom Jeon, Yejin Hong.

**Methodology:** Jungtae Leem, Hyunho Kim, Hee-Geun Jo, Tae-Hun Kim.

**Project administration:** Byungkwan Seo, Yeeun Cho, Ga Young Han.

**Resources:** Yeoncheol Park, Jung Won Kang.

**Supervision:** Dongwoo Nam.

**Writing – original draft:** Jungtae Leem.

**Writing – review & editing:** Jungtae Leem, Dongwoo Nam.
